# Dynamic contrast-enhanced breast MRI features correlate with invasive breast cancer angiogenesis

**DOI:** 10.1038/s41523-021-00247-3

**Published:** 2021-04-16

**Authors:** Jennifer Xiao, Habib Rahbar, Daniel S. Hippe, Mara H. Rendi, Elizabeth U. Parker, Neal Shekar, Michael Hirano, Kevin J. Cheung, Savannah C. Partridge

**Affiliations:** 1 Department of Radiology, University of Washington, Seattle, WA USA; 2 Breast Imaging, Seattle Cancer Care Alliance, Seattle, WA USA; 3 Department of Pathology, University of Washington, Seattle, WA USA; 4 Department of Medicine, Division of Medical Oncology, University of Washington, Seattle, WA USA; 5 Translational Research Program, Public Health Sciences and Human Biology Divisions, Fred Hutchinson Cancer Research Center, Seattle, WA USA

**Keywords:** Breast cancer, Cancer imaging, Tumour angiogenesis

## Abstract

Angiogenesis is a critical component of breast cancer development, and identification of imaging-based angiogenesis assays has prognostic and treatment implications. We evaluated the association of semi-quantitative kinetic and radiomic breast cancer features on dynamic contrast-enhanced (DCE)-MRI with microvessel density (MVD), a marker for angiogenesis. Invasive breast cancer kinetic features (initial peak percent enhancement [PE], signal enhancement ratio [SER], functional tumor volume [FTV], and washout fraction [WF]), radiomics features (108 total features reflecting tumor morphology, signal intensity, and texture), and MVD (by histologic CD31 immunostaining) were measured in 27 patients (1/2016–7/2017). Lesions with high MVD levels demonstrated higher peak SER than lesions with low MVD (mean: 1.94 vs. 1.61, area under the receiver operating characteristic curve [AUC] = 0.79, *p* = 0.009) and higher WF (mean: 50.6% vs. 22.5%, AUC = 0.87, *p* = 0.001). Several radiomics texture features were also promising for predicting increased MVD (maximum AUC = 0.84, *p* = 0.002). Our study suggests DCE-MRI can non-invasively assess breast cancer angiogenesis, which could stratify biology and optimize treatments.

## Introduction

Angiogenesis is a critical factor in the development and progression of breast cancers. Once primarily thought to be driven by genetic alterations of ductal epithelial cells, breast cancer tumorigenesis and metastasis are now recognized also to be dependent on microenvironment factors including basement membrane permeability to allow stromal invasion, local immune suppression to facilitate malignant cells evasion, and angiogenesis to supply nutrients for tumor growth and hematogenous spread^1^. Biologically, angiogenesis is initiated by hypoxic insult to the developing malignancy, which triggers activation of hypoxia inducible factor transcriptional activity (e.g. HIF-1), leading to vascular endothelial growth factor (VEGF) production and release^2^. In turn, VEGF initiates a cascade of endothelial cell proliferation, migration, tube formation, and maturation into microvessels^3^. Clinically, higher levels of angiogenesis are associated with worse patient outcomes in breast cancer^4–6^ and the development of distant metastasis^7^, which also suggests treatment possibilities with anti-angiogenic therapies^8^.

As angiogenesis is a dynamic in vivo process, its quantitation pathologically relies on indirect measures, including the surrogate marker of microvessel density (MVD). MVD is measured by counting small and tortuous vessels in the tumor tissue by immunohistochemical staining using antibodies such as factor VIII antigen (von Willebrand factor), CD31 and CD34^4,9,10^. High MVD has been shown to predict poor survival in women with invasive breast cancer, with especially high prognostic value in women who present with lymph node negative disease, and has been proposed as a marker to identify patients at high risk of recurrence^4^. High MVD is also associated with other aggressive features, including higher histologic grade and negative estrogen receptor status^11,12^ and has been shown to correlate with the occurrence of osseous metastases^13^. Although MVD assessments vary in technique, studies have shown that inter- and intra-reader variability can be low with appropriate training^14^.

Dynamic contrast-enhanced magnetic resonance imaging (DCE-MRI) is routinely utilized in patients with newly diagnosed breast cancer to assess the local extent of disease and assist in surgical and treatment planning. DCE-MRI probes the vascular environment over several timepoints after intravenous injection of a contrast agent, and the varying signal can be described using quantitative enhancement kinetic features. During routine interpretation, semi-quantitative enhancement kinetic features are often utilized to improve specificity for identifying malignancy, including initial peak enhancement and the presence of delayed phase washout, as defined by the American College of Radiology (ACR) Breast Imaging-Reporting And Data System (BI-RADS) Breast MRI Atlas^15–17^. These semi-quantitative kinetics features have been hypothesized to reflect tumor angiogenesis^9,18,19^, though there is a paucity of literature supporting this directly. Additionally, recent advances in computational image analysis have given rise to the new field of “radiomics”, where a high dimensional panel of quantitative imaging features are extracted from routine diagnostic radiology studies, which further expands the potential to utilize DCE-MRI to non-invasively characterize underlying tumor architecture, biology, and function^20,21^.

Thus, we sought to assess the association of breast DCE-MRI enhancement features with angiogenesis using semi-quantitative kinetic measurements and radiomics analyses. Specifically, we hypothesized that MRI parameters that reflect early delivery of contrast [initial peak percent enhancement (peak PE)], rapid clearance of contrast [peak signal enhancement ratio (peak SER) and washout fraction (WF)], and greater functional tumor volume (FTV) would correlate with higher levels of MVD. As an exploratory aim, we sought to assess the relationships between comprehensive radiomics analysis-based features of intratumoral heterogeneity and texture with MVD.

## Results

### Patient cohort and characteristics

A total of 33 patients with a diagnosis of invasive breast cancer consented to participate in the study and underwent routine DCE-MRI assessment as well as additional pathologic assessment of MVD. Six patients were subsequently excluded from the study due to either obscuration of MRI findings by post-biopsy changes (*n* = 4) or unavailable pathologic (*n* = 2) specimens, leaving a final cohort of 27 patients (median age = 53 years, range = 30 to 88) with complete data. Median tumor size was 23 mm (range 10–101), with 23 lesions diagnosed as invasive ductal carcinoma (IDC), three invasive lobular carcinoma (ILC), and one invasive mammary carcinoma not otherwise specified. Median MVD was 17 (range 8–56) microvessels per 0.152 mm^2^ (×400) field, with 13 invasive cancers categorized as high MVD and 14 low MVD. Other histopathologic characteristics, including Nottingham Grade, Ki-67, hormone receptor status, human epidermal growth factor 2 (HER2) status, and T stage are summarized in Table 1. None of these patient and tumor characteristics differed significantly between MVD groups (*p* > 0.05 for each) (Supplementary Table 1).

**Table 1:** Patient and tumor characteristics.

Variable	Value
Age, years	53 (30-88)
Histology	
Invasive ductal carcinoma	23 (85)
Invasive lobular carcinoma	3 (11)
Invasive mammary carcinoma	1 (4)
Nottingham grade	
1	7 (26)
2	7 (26)
3	13 (38)
Ki-67	
High	14 (52)
Low	9 (33)
Not available	4 (15)
Estrogen receptor	
Positive	20 (74)
Negative	7 (26)
Progesterone receptor	
Positive	20 (74)
Negative	7 (26)
HER2	
Positive	9 (33)
Negative	18 (67)
Lymph Node Status	
N0	12 (44)
N1+	15 (56)
T stage	
I	8 (30)
II	13 (48)
III	4 (15)
IV	2 (7)
Microvessel density	
Low	14 (52)
High	13 (48)

Values are median (range) or no. (%). *HER2* human epidermal growth factor receptor 2.

### Associations between kinetic parameters and MVD

**Fig. 1:**
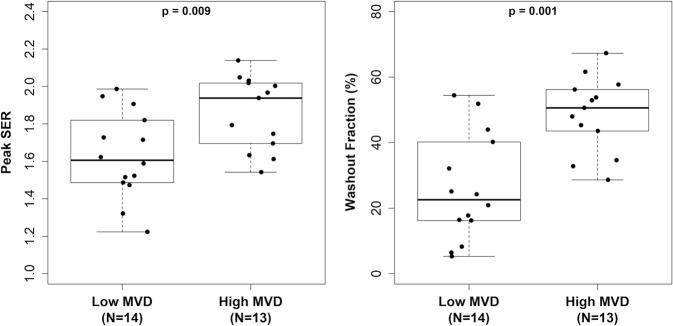
DCE-MRI kinetics features associated with microvessel density. Two kinetics features significantly differentiated lesions with low versus high microvessel density (MVD). Box-and-whisker plots represent the median (solid line), inter-quartile range (IQR; box), and range of the dynamic contrast-enhanced (DCE)-MRI metrics. The whiskers extend up to 1.5 times the IQR from the box to the smallest and largest points. Lesions with higher MVD based on CD31 staining on average demonstrated higher peak signal enhancement ratio (SER; AUC: 0.79, *p* = 0.009, left) and higher washout fraction (AUC: 0.87, *p* = 0.001, right).

**Fig. 2:**
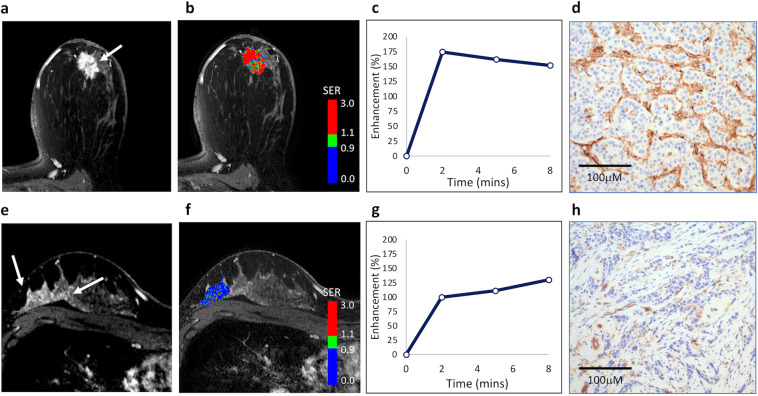
DCE-MRI characteristics in two malignancies with different levels of microvessel density. Top row: a 44-year old woman with biopsy-proven grade II invasive ductal carcinoma. **a** DCE-MRI demonstrates an enhancing lesion in the subareolar region of the left breast (arrow). **b** Kinetics analysis shows rapid contrast enhancement and washout, with predominantly high signal enhancement ratio (SER), with peak SER = 2.1 (initial peak percent enhancement [PE] = 310%, functional tumor volume [FTV] = 4.9 cm^3^, and washout fraction [WF] = 56%; red indicates areas of washout [SER ≥ 1.1]). **c** Plot of overall lesion enhancement (%) versus time after contrast injection demonstrates high early peak enhancement and washout by 8 min. **d** Histologic staining for CD31 indicates high microvessel density (MVD; shown at ×400 magnification), with mean vessel count = 56. Bottom row: a 34-year old woman with biopsy-proven grade III invasive ductal carcinoma. **e** DCE-MRI demonstrates an enhancing lesion in the posterior right breast. **f** Kinetics analysis shows predominantly low SER, with peak SER = 1.3 (initial peak PE = 218%, FTV = 4.8 cm^3^, and WF = 6%)), with mean vessel count = 9. **g** Plot of overall lesion enhancement (%) versus time after contrast injection demonstrates persistent enhancement over 8 min. **h** Histologic staining for CD31 indicates low MVD (shown at ×400 magnification).

Both peak SER and WF kinetic features were positively associated with MVD. Compared with counterparts with low MVD, invasive breast cancers with high MVD exhibited higher peak SER values (median: 1.94 vs. 1.61, area under the receiver operating characteristic curve [AUC] = 0.79, *p* = 0.009) and higher WF (median: 50.6% vs. 22.5%, AUC = 0.87, *p* = 0.001), which were statistically significant at the Bonferroni-corrected α = 0.0125 (Fig. 1). However, there was no significant relationship between initial peak PE (median: 214% vs. 199%, AUC = 0.61, *p* = 0.35) or FTV (median: 3.0 vs. 4.7 cm^3^, AUC = 0.60, *p* = 0.40) and MVD (Table 2). As a sensitivity analysis, the semi-quantitative kinetic parameters were also compared with continuous MVD counts. Consistent with the primary analysis, peak SER (Spearman’s *r* = 0.55, *p* = 0.003) and WF (Spearman’s *r* = 0.60, *p* = 0.001) were significantly correlated with higher MVD on a continuous basis (Supplementary Table 2). Examples of two lesions with high and low MVD are shown in Fig. 2.

**Table 2:** Univariable associations of kinetic parameters with MVD.

	MVD^a^						
	Low	High					
Variable	(*N* = 14)	(*N* = 13)	OR^b^	(95% CI)	AUC	(95% CI)	*P*
Peak PE (%)	199 (133, 258)	214 (106, 332)	1.4	(0.6, 3.2)	0.61	(0.38, 0.84)	0.35
Peak SER	1.61 (1.22, 1.99)	1.94 (1.54, 2.14)	3.3	(1.2, 9.2)	0.79	(0.62, 0.96)	0.009
FTV (cm^3^)	4.7 (0.4, 93.6)	3.0 (0.3, 85.2)	0.8	(0.4, 1.9)	0.60	(0.38, 0.82)	0.40
Washout fraction (%)	22.5 (5.3, 54.4)	50.6 (28.6, 67.3)	6.7	(1.7, 25.7)	0.87	(0.73, 1.00)	0.001

*AUC* area under the receiver operating characteristic curve, *CI* confidence interval, *FTV* functional tumor volume, *MVD* microvessel density, *OR* odds ratio, *PE* percent enhancement, *SER* signal enhancement ratio.

^a^Values are median (range).

^b^Change per 1-SD increase in variable.

### Associations of radiomic analysis with MVD

**Fig. 3:**
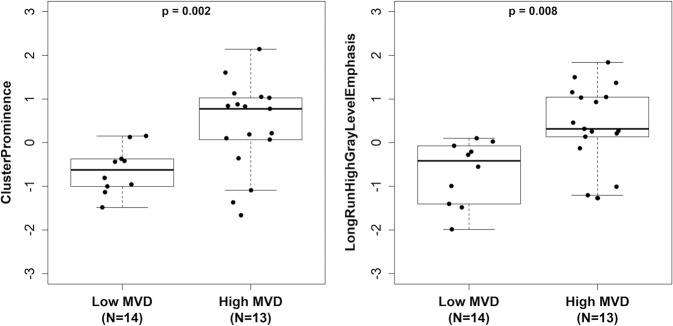
DCE-MRI radiomics features most associated with microvessel density. Two radiomic texture features that significantly differentiated lesions with low versus high microvessel density (MVD). Box-and-whisker plots represent the median (solid line), inter-quartile range (IQR; box) and range of the radiomics features. The whiskers extend up to 1.5 times the IQR from the box to the smallest and largest points. Radiomics features were log-transformed to reduce skewness and standardized to have a standard deviation equal to 1. Lesions with higher MVD based on CD31 staining on average demonstrated higher GLCM—ClusterProminence values (AUC: 0.84, *p* = 0.002, left) and higher GLRLM—LongRunHighGrayLevelEmphasis values (AUC: 0.83, *p* = 0.003, right).

**Fig. 4:**
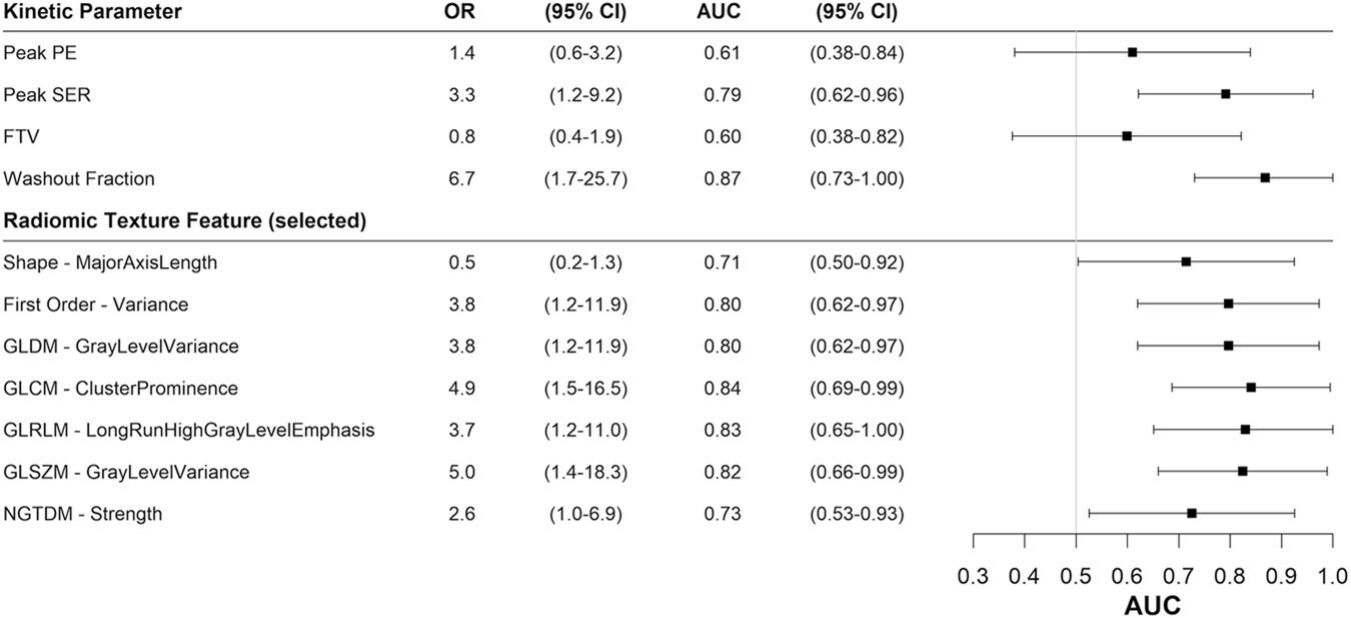
Performance of DCE-MRI kinetics and radiomics features for distinguishing lesions with different microvessel densities. Odds ratios (ORs) and forest plot of area under the receiver operating characteristic curve (AUC) for predicting high microvascular density (MVD) are shown for all kinetic parameters and selected radiomic texture features. ORs are scaled to correspond to differences per 1-standard deviation increase. The error bars represent 95% confidence intervals (CIs). The texture feature with the strongest association with MVD in terms of AUC is shown for each radiomic feature category. See Table 3 and Supplementary Table 2 for more detail.

Exploratory analysis of the association of radiomic features revealed multiple features to be potentially associated with MVD, including several GLDM features, GLCM features and GLRLM features (*p* < 0.01), as shown in Table 3. Results for all radiomics features are shown in Supplementary Table 3 and Supplementary Table 4. Two texture-based radiomics feature in particular had large estimated associations with MVD: GLCM-ClusterProminence (AUC = 0.84, *p* = 0.002), which measures skewness and asymmetry of the GLCM (a matrix describing the distribution of neighboring voxel gray levels), and GLRLM-LongRunHighGrayLevelEmphasis (AUC = 0.83, *p* = 0.003), which measures the joint distribution of long run lengths with higher gray-level values (Fig. 3). Relative performance of kinetics and radiomics features for differentiating lesions with low versus high MVD (odds ratios and AUCs) is shown in Fig. 4.

**Table 3:** Exploratory univariable analysis of associations between MVD and selected^a^ DCE-MRI radiomic texture features.

	OR^b^	(95% CI)	AUC	(95% CI)	*P*
Shape features					
MajorAxisLength	0.5	(0.2, 1.3)	0.71	(0.50, 0.92)	0.061
First-order features					
Variance	3.8	(1.2, 11.9)	0.80	(0.62, 0.97)	0.008
GLDM features					
GrayLevelVariance	3.8	(1.2, 11.9)	0.80	(0.62, 0.97)	0.008
GLCM features					
ClusterShade	0.2	(0.0, 0.8)	0.80	(0.62, 0.97)	0.008
ClusterProminence	4.9	(1.5, 16.5)	0.84	(0.69, 0.99)	0.002
GLRLM features					
GrayLevelVariance	3.9	(1.2, 12.6)	0.80	(0.63, 0.98)	0.007
LongRunHighGrayLevelEmphasis	3.7	(1.2, 11.0)	0.83	(0.65, 1.00)	0.003
RunEntropy	3.9	(1.2, 13.2)	0.80	(0.62, 0.97)	0.008
GLSZM features					
GrayLevelVariance	5.0	(1.4, 18.3)	0.82	(0.66, 0.99)	0.003
GrayLevelNonUniformityNormalized	0.2	(0.1, 0.8)	0.79	(0.62, 0.97)	0.009
NGTDM features					
Strength	2.9	(0.9, 9.5)	0.73	(0.53, 0.93)	0.048

*AUC* area under the receiver operating characteristic curve, *CI* confidence interval, *GLCM* gray-level contrast matrix, *GLDM* gray-level difference matrix, *GLRLM* gray-level run length matrix, *GLSZM* gray-level size zone matrix, *NGTDM* neighborhood gray tone difference matrix, *OR* odds ratio.

^a^Features shown are those with *p* < 0.01 or with the largest association in each feature category in terms of AUC. See Supplemental Table 3 and 4 for results of all features tested.

^b^Change per 1-SD increase in variable.

## Discussion

In this single-institution study of women diagnosed with invasive breast cancer, we found that tumors with higher MVD exhibited more rapid clearance of contrast as reflected by peak SER and WF on DCE-MRI. In an exploratory analysis, we found multiple promising radiomic features that may also predict MVD levels, with GLCM- ClusterProminence demonstrating the highest association. Our findings suggest that semi-quantitative DCE-MRI features correlate with tumor angiogenesis as measured by MVD and could potentially map to other molecular steps of the angiogenesis cascade^22^. In this way, semi-quantitative DCE-MRI offers the potential for multiparametric, simultaneous, and non-invasive assessment of the angiogenic process in its native context. Our results may also be translatable to clinical practice as our study utilized a standard ACR accredited clinical MRI, which emphasizes spatial rather than temporal resolution as with many research protocols. WF in particular, is routinely reported on commercially available computer-aided evaluation software tools (e.g. CADstream, Merge Healthcare Inc, Chicago, IL) allowing for straightforward implementation in clinical practice.

Extending on the clinical implications of these findings, semi-quantitative DCE-MRI could hold additional prognostic potential, particularly in the neoadjuvant setting. In principle, anti-angiogenic therapy could limit tumor growth and normalize vasculature, thereby improving delivery of neoadjuvant therapy^22–24^. However, clinical trials conducted over the last decade in unselected breast cancer patients have not shown increased overall survival with the addition of anti-angiogenic therapy, despite increased pCR in some studies^2,25–27^. At present, there are parallel efforts to identify biologically based high-risk subgroups benefiting from anti-angiogenic targeting, including inflammatory breast cancers^28^, metaplastic subtype^29^, and in triple negative breast cancers in BRCA1/2 mutation carriers^30^. In addition, there is sustained interest in combining anti-angiogenesis agents with immune checkpoint blockade^31–33^. Vascular normalization could have particular relevance in the triple negative breast cancer setting where the addition of pembrolizumab to neoadjuvant chemotherapy has been shown to increase pathologic complete response^34^. Our study suggests that semi-quantitative DCE-MRI could provide a non-invasive method for querying the angiogenic microenvironment in these clinically important subgroups, and could complement other molecular approaches for biomarker development including circulating growth factors, metabolic proteins, and hypoxia molecular signatures^35–38^.

Previous studies exploring the association with DCE-MRI and MVD have demonstrated somewhat variable results^39–45^. Similar to our findings, Esserman et al found in their study of 32 patients that individual SER values were significantly correlated with intratumoral MVD hot spots^39^. Poellinger et al also demonstrated positive associations with early contrast enhancement (initial peak PE) and MVD^40^. However, multiple other studies did not find a correlation with MVD and DCE-MRI kinetic features, although these studies differed significantly in their technique, most notably with regards to MRI acquisition protocols^16,41–43^. A recent investigation by Mori et al specifically focused on utilization of an alternative ultrafast DCE-MRI protocol to assess enhancement kinetics^41^. Interestingly, they found correlation of several ultrafast semi-quantitative kinetic parameters with MVD, most notably initial slope of enhancement, but did not find any such correlation using standard DCE timings and parameters including SER. Ultimately, the ability of kinetics-based measures to serve as meaningful biomarkers may be limited by varying technical approaches specific to each site, and are therefore likely to be less robust and translatable.

Radiomics analysis has been touted to circumvent the issue of inter-platform variability and may offer more reproducible results compared to semi-quantitative and/or quantitative kinetic features. This is because radiomics features can be extracted utilizing a standardized computational approach^46^ based on data acquired at a single timepoint, whereas traditional kinetic features rely heavily on arterial input functions and imaging timings, which may contribute to conflicting results such as those discussed above. Multiple early studies have demonstrated promise for radiomics features to correlate with prognostic factors of breast cancers, including treatment response, genomic assays of recurrence risk, and level of immune activation^47–51^. Few limited studies have explored associations of radiomics features with MVD, and to our knowledge only in non-breast malignancies, such as prostate cancer and renal cell carcinoma. These studies demonstrated promising results with several gray level and wavelet features correlating with MVD^52,53^. Our study found that of radiomic features, GLCM and GLRLM features, which primarily reflect spatial coherence and connectivity between voxels, most significantly correlated with MVD in breast cancers. Similarly, Yin et al also found several GLRLM features correlating with MVD in renal cell carcinomas^53^. Future work is needed to determine whether these relationships between radiomics features and MVD or other tumor angiogenesis markers persist in larger studies and across tumor types.

Another potential explanation for varying results between studies is variation in MVD assessment. Although most studies including our study used a similar technique by counting microvessels at the highest areas of neovascularization, there is no universally recommended immunostaining for MVD. In our study, MVD was measured using immunostaining with CD31, whereas several other studies utilized either factor VIII antigen or CD34. Both CD31 and CD34 have been shown to be more sensitive compared to factor VIII antigen in the assessment of MVD due to staining of isolated endothelial cells and immature vessels in addition to larger microvessels^54^. However, CD31 can react mildly with inflammatory cells and is rarely expressed strongly, with staining failure rates of up to 20%^4,45^. Some studies suggest CD34 may be superior to CD31 as it is more strongly expressed on microvessels^4,54^.

Higher levels of MVD have been shown to predict poor survival and correlate with aggressive subtypes of breast cancer, such as higher histologic grade and negative estrogen receptor status^4,11,12^. However, MVD assessment is known to have inter-reader variability, with several different studies attempting to minimize its subjectivity^4,14^. In prior studies, MVD is most commonly assessed as a binary variable, with many studies using the median value as the cutoff^4^. Interestingly, there are no standard published cutoff values for high MVD. In our study, we therefore assessed MVD as both a continuous and categorical variable.

While our study demonstrated a correlation of higher peak SER and WF with increased MVD, we did not identify initial peak PE and FTV to be similarly related to MVD. This likely results from the fact that early tumor enhancement in the first several minutes (from which both initial peak PE and FTV metrics are calculated) is determined by multiple additional factors beyond MVD. Specifically, initial peak PE is believed to be affected by blood flow, in addition to technical aspects such as contrast dose and injection rate^55^, and other patient factors such as cardiac function. Furthermore, FTV is also primarily weighted by tumor size and may be less likely to demonstrate a direct relationship with MVD. In contrast, SER and WF are metrics that both reflect the degree of washout from the tumor, which we now posit is more directly related to the density of leaky and abnormal vessels that have been recruited to supply an aggressive malignancy and are less dependent on other factors.

Our study has several important limitations. First, we utilized CD31 for immunostaining of MVD, which as noted above may be less sensitive when compared to CD34. Second, our study did not include benign lesions for comparison, and thus we cannot directly determine that imaging features’ associations with MVD have clear diagnostic value. Third, our sample size was relatively small, which limited our ability to assess the relative value of the different DCE-MRI radiomics metrics and perform multivariable analyses. Fourth, our study explored semi-quantitative rather than quantitative kinetic parameters. Quantitative measurements such as Ktrans, Vp, and Kep were not included because our study used clinical DCE-MRI scans, which emphasizes spatial resolution over temporal resolution and does not allow for pharmacokinetic mapping. Most pharmacokinetic mapping breast MRI studies sacrifice coverage or spatial resolution for rapid temporal sampling and are not as amenable for clinical interpretation or radiomics analysis. Finally, our analysis of texture features was hypothesis-generating and did not fully adjust for multiple comparisons. While some features appeared to have promising performance at discriminating between higher and lower MVD, these findings should be interpreted with caution and further studies in larger cohorts are warranted.

In conclusion, our study demonstrates that semi-quantitative DCE-MRI features have promise to serve as markers of MVD in invasive breast cancers, and could potentially identify additional non-invasive features mapping to other molecular steps of the angiogenesis cascade. Furthermore, our findings suggest that radiomics features may provide an even more robust approach to non-invasively evaluating MVD. If validated in larger studies, these features could provide prognostic value and aid in identifying patient subgroups who may benefit from anti-angiogenic therapy and also complement other molecular approaches for biomarker development.

## Methods

### Patients

This prospective study was approved by the Institutional Review Board of the Fred Hutchinson Cancer Research Center (IR#8148) and was compliant with the Health Insurance Portability and Accountability Act. All patients provided informed consent and were enrolled at the University of Washington/Seattle Cancer Care Alliance (SCCA) between January 2016 and July 2017. Patients over the age of 18 were eligible for the study if they had a new diagnosis of stage I - III invasive breast cancer with a minimum lesion size of 1.5 cm identified on core needle biopsy and underwent a clinical breast MRI at SCCA to evaluate the extent of disease. Patients were not eligible if they were treated with chemotherapy before the MRI or surgical treatment of the cancer, as this could affect both the MRI and pathology features measured.

### MRI Acquisition

The MRI protocols followed guidelines established by the American College of Radiology breast MRI accreditation program^56^. The protocol included a 3D T1-weighted fast gradient echo-based DCE series with one pre and three sequential post-gadolinium contrast-enhanced sequences with scan durations of approximately 3 min each. All scans were performed on a 3 Tesla Philips Achieva Tx system (Philips Medical Systems, Best, the Netherlands) with a 16-channel breast coil (Mammotrak, Philips Healthcare). DCE-MRI was performed using a fat saturated 3D fast gradient echo sequence (eTHRIVE: enhanced T1-weighted High Resolution Isotropic Volume Excitation) with the following parameters: TR/TE = 5.9/3 msec, flip angle = 10°, spatial resolution = 0.5 × 0.5 × 1.3 mm. Post-contrast sequences were acquired with k space centered at approximately 120, 300, and 480 s after contrast injection. All scans were acquired in the axial orientation. The contrast agent administered was 0.1 mmol/kg body weight gadoteridol (ProHance, Bracco Diagnostics, Milan, Italy) at the rate of 2 cc/s followed by a 15 cc saline flush.

### DCE-MRI image analysis

All MR image analyses were performed with observers blinded to pathologic outcomes, using customized open source software tools for kinetic enhancement measurements and radiomic texture measurements as detailed below.

#### Kinetic Analysis

DCE-MRI imaging volumes were first co-registered using a commercially available computer-aided evaluation software tool (CADstream, Merge Healthcare Inc, Chicago, IL) to correct for any misregistration due to patient motion between pre and post-contrast acquisitions. Next, semiautomated tumor segmentation and quantitation were performed by imaging researchers (N.S., M.H.) under the guidance of a radiologist (H.R., 10 years experience breast imaging) and imaging scientist (S.C.P., 15 years experience in quantitative breast MRI) who reviewed all tumor segmentations. Tumors were segmented on DCE-MRI using a custom software tool developed in MATLAB (MathWorks, Natick, MA) to select enhancing voxels and define a 3D region of interest. Kinetics analysis was then performed using semiautomated software written in-house in Java language and ImageJ (NIH, public domain, Bethesda, MD). Contrast kinetics were characterized by several parameters as previous described^57,58^: (1) Initial PE, (2) SER, (3) FTV, and (4) WF. Initial PE (units, %) reflects the degree of signal enhancement in the tumor at 120 s after contrast delivery, calculated by

1 $${\rm{PE}} = \frac{{S_1 - S_0}}{{S_0}} \times 100$$

where *S*_0_ is the MRI signal intensity prior to contrast and *S*_1_ is the MRI signal intensity 120 s after contrast delivery. SER is a unitless index that reflects the rate of contrast washout in the tumor between 120 and 480 s after contrast delivery, calculated by:

2 $${\rm{SER}} = \frac{{S_1 - S_0}}{{S_2 - S_0}}$$

where *S*_2_ is the MRI signal intensity at 480 s after contrast delivery. Initial PE and SER were calculated on a voxel-by-voxel basis within the tumor, with SER calculated only for voxels with at least 50% initial PE. To determine peak PE and peak SER, the software automatically identified independent hot-spot tumor regions of 3 × 3 × 3 voxels (0.1625 mm^3^) producing the highest mean initial PE and SER value, respectively. FTV (units, cm^3^) was calculated by summing volumes for all voxels with initial PE ≥ 50%. Additionally, WF percentage was calculated as the fraction of all tumor voxels exhibiting washout (defined as those with PE ≥ 50% and SER ≥ 1.1).

**Fig. 5:**
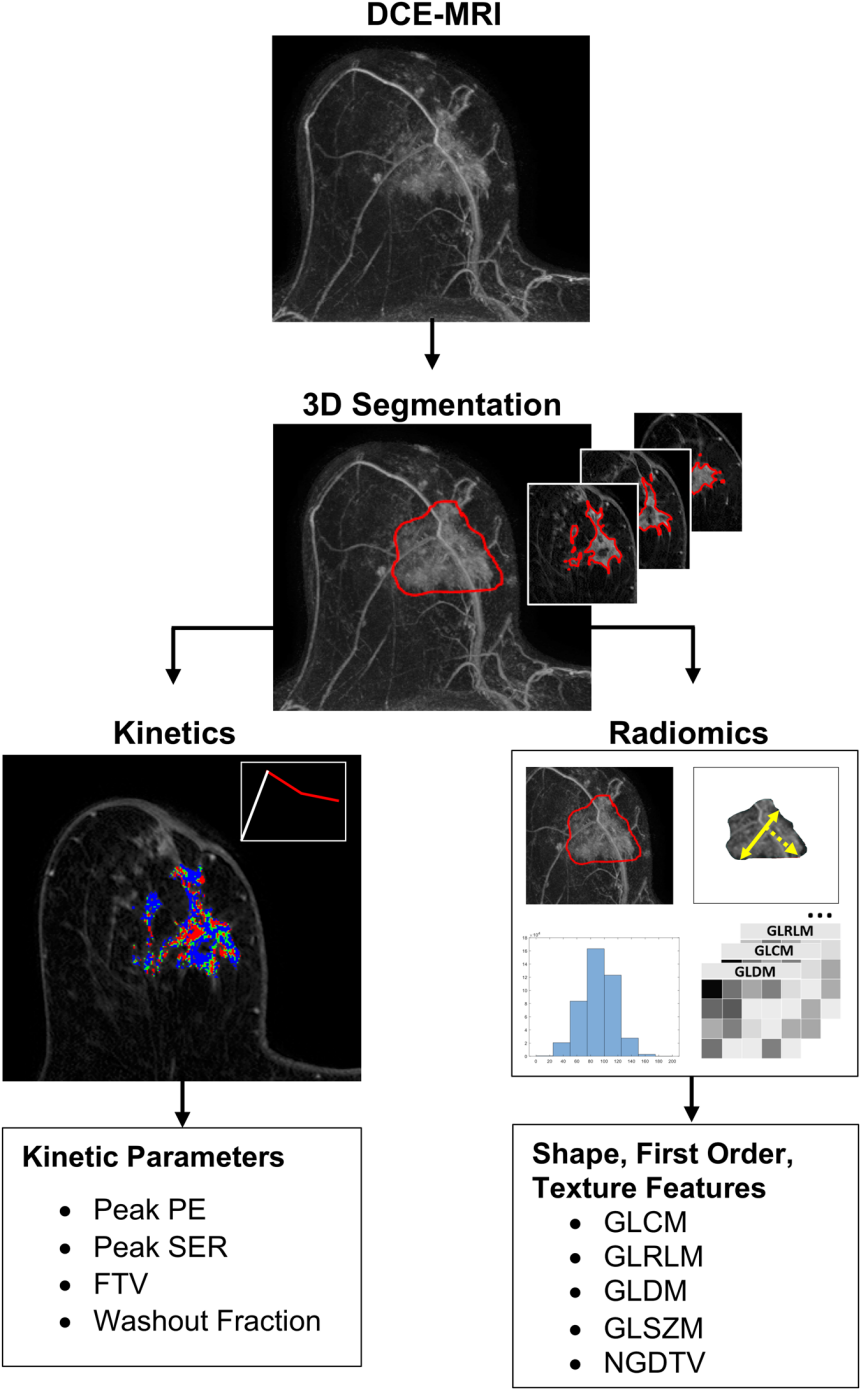
Postprocessing pipeline for quantitative DCE-MRI lesion assessment. Both kinetics and radiomics parameters are extracted for three-dimensional segmented tumor volumes (illustrated in 62-year old woman with biopsy proven invasive ductal carcinoma). DCE-MRI dynamic contrast-enhanced magnetic resonance imaging, PE percent enhancement, SER signal enhancement ratio, FTV functional tumor volume, GLCM gray-level contrast matrix, GLRLM gray-level run length matrix, GLDM gray-level difference matrix, GLSZM gray-level size zone matrix, NGTDM neighborhood gray tone difference matrix.

#### Radiomic analysis

The 3D segmented lesions were also evaluated using an open source software platform for radiomics analyses (3D Slicer v4.11.0 with pyradiomics library^59^, www.slicer.org) of the initial contrast-enhanced DCE-MR images (120 s post-contrast). Each DCE-MRI dataset (volumetric stack) was normalized to have zero mean and unit variance, and isotropic resampling in 3 dimensions was applied before the radiomics features were calculated. A total of 108 standardized radiomics features^46^ were generated on each tumor volume for exploratory statistical analysis, within the following feature categories: (1) first-order histogram statistics based on voxel signal intensities (gray levels), (2) shape-based descriptors, (3) gray level co-occurrence matrix (GLCM) based on the second-order joint probability functions of voxel intensities in a particular spatial region, (4) gray level size zone matrix (GLSZM), quantifying gray level zones as number of connected voxels with the same gray level intensity, (5) gray level run length matrix (GLRLM), quantifying gray level runs as length (number of consecutive pixels) with the same gray level value, (6) gray level dependence matrix (GLDM), quantifying the number of connected voxels in a given distance that are dependent on the center voxel, and (7) neighboring gray tone difference matrix (NGTDM), quantifying differences in gray levels of neighboring voxels. The MR imaging postprocessing pipeline is summarized in Fig. 5.

### Pathologic assessment

All biopsy and surgical specimens pathologically assessed at our institution include verification of invasive cancer, tumor grade (by Nottingham histologic score reflecting tumor cell differentiation based on tubule formation, nuclear grade, and mitotic rate), ER and PR status (by Allred score for expression), HER2 status (positive or negative by immunohistochemistry and/or fluorescence in situ hybridization), and Ki-67 proliferation index. For the study, MVD was assessed by immunostaining with mouse antihuman CD31 monoclonal antibody (JC/70A) on a single representative tumor specimen slide obtained from the surgical tissue block (or from the diagnostic core needle biopsy tissue block in patients who underwent chemotherapy treatment before their surgery). The two most vascularized areas within the tumor (‘hot spots’) were chosen at low magnification (×40) and vessels were counted in a representative high magnification (×400; 0.152 mm^2^; 0.44 mm diameter) field in each of these two areas. Single immunoreactive endothelial cells, or endothelial cell clusters separate from other microvessels, were counted as individual microvessels. Endothelial staining in large vessels with tunica media, and nonspecific staining of nonendothelial structures, were disregarded in microvessel counts. The MVD counts from the two microscopic fields per specimen were averaged. Two board certified anatomic pathologists specializing in breast pathology (M.H.R. 10 years, E.U.P. two years sub-specialty experience) independently reviewed the same CD31-stained slides to assess MVD as described, blinded to the clinical and imaging data. MVD counts from the two pathologists were averaged for the statistical analysis, except those where the difference was large (≥ 12), which were adjudicated by reviewing the case together to reach consensus.

### Statistical analyses

Continuous variables were summarized as median (range) and categorical variables as number (percent). For the primary analysis, univariable associations were assessed between kinetic parameters and MVD dichotomized as low (≤ median value) vs. high (> median value). Associations were summarized using odds ratios (ORs) from univariable logistic regression models and the area under the receiver operating characteristic curve (AUC). Differences between lower and higher MVD were tested using the Wilcoxon rank-sum test. Due to testing four kinetic parameters, we used a Bonferroni-corrected significance threshold of α = 0.0125 to determine statistical significance. As a sensitivity analysis, we also performed an analogous analysis where we used Spearman’s rank correlation coefficient to evaluate associations of kinetic parameters with MVD as a continuous variable.

As a secondary, hypothesis-generating analysis, we used similar methods to evaluate associations of radiomic texture features with MVD, both as a dichotomous variable and a continuous variable. We used a significance threshold α = 0.01 to reduce the risk of false positive findings, though did not perform a full adjustment for multiple comparisons to avoid a substantial loss of statistical power. Two-sided confidence intervals (CIs) were calculated for all ORs, AUC values, and Spearman’s rank correlation coefficients. A confidence level of 95% was used for all CIs. All statistical tests performed were two-sided. Statistical calculations were conducted with the statistical computing language R (version 3.1.1; R Foundation for Statistical Computing, Vienna, Austria).

### Reporting summary

Further information on research design is available in the Nature Research Reporting Summary linked to this article.

## Supplementary information

Supplementary Information

Reporting Summary

## Data Availability

The data generated and analyzed during this study are described in the following figshare data record: 10.6084/m9.figshare.13574570^60^. The de-identified DCE-MRI data, measures and metadata will be shared as part of the figshare data record. Deidentified MR images will be available upon request, and interested parties should contact the corresponding author. The pathology CD-31 stained slides data are not publicly available for the following reason: no digitized versions of the slides have been created.

## Code availability

The open source software platform 3D Slicer (v4.11.0 with pyradiomics library, www.slicer.org) was utilized for radiomics analysis as referenced in the methods section. Software for lesion segmentation and kinetics analysis were developed in-house using MATLAB (MathWorks, Natick, MA) and the open source software platform ImageJ (NIH, public domain, Bethesda, MD), respectively, and can be made available upon request to the corresponding author.
