# Supplementary material for: Dynamic contrast-enhanced breast MRI features correlate with invasive breast cancer angiogenesis
Source: NPJ Breast Cancer. 2021 Apr 16;7:42. doi: 10.1038/s41523-021-00247-3 (PMC8052427; doi:10.1038/s41523-021-00247-3)
Supplement: Supplementary file 1 — Supplementary Information [file 41523_2021_247_MOESM1_ESM.pdf]

Supplementary Table 1. Comparison of patient and tumor characteristics between MVD groups.

Variable	MVD* Low (N=14)	MVD* High (N=13)	P-value†
Age, years	54 (30 - 88)	52 (34 - 72)	0.81
Histology			0.22
Invasive Ductal Carcinoma	11 (79)	12 (92)	
Invasive Lobular Carcinoma	3 (21)	0 (0)	
Invasive Mammary Carcinoma	0 (0)	1 (8)	
Nottingham Grade			0.61
1	5 (36)	2 (15)	
2	3 (21)	4 (31)	
3	6 (43)	7 (54)	
Ki-67**			>0.99
High	8 (57)	6 (67)	
Low	6 (43)	3 (33)	
Estrogen Receptor			0.68
Positive	3 (21)	4 (31)	
Negative	11 (79)	9 (69)	
Progesterone Receptor			0.68
Positive	3 (21)	4 (31)	
Negative	11 (79)	9 (69)	
HER-2			0.24
Positive	11 (79)	7 (54)	
Negative	3 (21)	6 (46)	
Lymph Node Status			>0.99
N0	6 (43)	6 (46)	
N1+	8 (57)	7 (54)	
T Stage			0.78
I	3 (21)	5 (38)	
II	7 (50)	6 (46)	
III	3 (21)	1 (8)	
IV	1 (7)	1 (8)	

*Values are median (range) or no. (%);

†Wilcoxon rank-sum test or Fisher's exact test comparison the MVD groups.

**Ki-67 was not available for four patients.

Supplementary Table 2. Univariable associations of kinetic parameters with MVD, based on both categorical and continuous MVD assessments.

	High MVD vs. Low MVD					Correlation with Continuous MVD		
Kinetic Parameters	OR*	(95% CI)	AUC	(95% CI)	P	Rho†	(95% CI)	P
Peak PE	1.4	(0.6, 3.2)	0.61	(0.38, 0.84)	0.35	0.28	(-0.14, 0.62)	0.15
Peak SER	3.3	(1.2, 9.2)	0.79	(0.62, 0.96)	0.009	0.55	(0.19, 0.79)	0.003
FTV	0.8	(0.4, 1.9)	0.60	(0.38, 0.82)	0.40	-0.18	(-0.47, 0.14)	0.37
Washout fraction	6.7	(1.7, 25.7)	0.87	(0.73, 1.00)	0.001	0.60	(0.24, 0.84)	0.001

Abbreviations: AUC = area under the receiver operating characteristic curve; CI = confidence interval; OR = odds ratio; PE = peak enhancement; SER = signal enhancement ratio;
*Change per 1-SD increase in variable;
†Spearman's rank correlation coefficient.

Supplementary Table 3. Univariable associations of radiomic texture features (shape, first order, and GLCM) with MVD, based on both categorical and continuous MVD assessments.

	High MVD vs. Low MVD					Correlation with Continuous MVD		
Shape Features	OR*	(95% CI)	AUC	(95% CI)	P	Rho†	(95% CI)	P
VoxelVolume	0.8	(0.3, 1.8)	0.57	(0.34, 0.80)	0.55	-0.19	(-0.51, 0.14)	0.34
Maximum3DDiameter	0.6	(0.2, 1.4)	0.69	(0.47, 0.90)	0.10	-0.41	(-0.69, -0.05)	0.034
MeshVolume	0.8	(0.3, 1.8)	0.57	(0.34, 0.80)	0.55	-0.19	(-0.51, 0.14)	0.34
MajorAxisLength	0.5	(0.2, 1.3)	0.71	(0.50, 0.92)	0.061	-0.44	(-0.70, -0.12)	0.020
Sphericity	1.6	(0.7, 3.7)	0.68	(0.45, 0.91)	0.13	0.33	(-0.05, 0.64)	0.091
LeastAxisLength	0.7	(0.3, 1.6)	0.59	(0.36, 0.82)	0.46	-0.21	(-0.52, 0.15)	0.29
Elongation	1.5	(0.7, 3.5)	0.63	(0.41, 0.86)	0.26	0.28	(-0.12, 0.63)	0.16
SurfaceVolumeRatio	0.6	(0.2, 1.3)	0.64	(0.42, 0.86)	0.22	-0.19	(-0.53, 0.19)	0.35
Maximum2DDiameterSlice	0.6	(0.2, 1.4)	0.68	(0.46, 0.89)	0.13	-0.36	(-0.65, 0.00)	0.062
Flatness	2.0	(0.8, 5.2)	0.65	(0.43, 0.87)	0.20	0.33	(-0.13, 0.65)	0.094
SurfaceArea	0.7	(0.3, 1.7)	0.64	(0.41, 0.87)	0.22	-0.31	(-0.64, 0.06)	0.11
MinorAxisLength	0.6	(0.2, 1.5)	0.70	(0.48, 0.92)	0.085	-0.39	(-0.69, -0.02)	0.044
Maximum2DDiameterColumn	0.6	(0.2, 1.6)	0.69	(0.48, 0.91)	0.094	-0.40	(-0.68, -0.05)	0.040
Maximum2DDiameterRow	0.5	(0.2, 1.4)	0.68	(0.46, 0.89)	0.13	-0.38	(-0.67, -0.02)	0.052
First Order Features								
InterquartileRange	3.4	(0.9, 12.4)	0.73	(0.53, 0.92)	0.048	0.45	(0.13, 0.68)	0.018
Skewness	0.4	(0.1, 1.0)	0.68	(0.47, 0.89)	0.12	-0.30	(-0.62, 0.12)	0.13
Uniformity	0.4	(0.2, 1.0)	0.71	(0.52, 0.91)	0.061	-0.41	(-0.66, -0.10)	0.031
Median	2.5	(1.0, 6.5)	0.73	(0.53, 0.93)	0.043	0.36	(0.02, 0.64)	0.064
Energy	1.1	(0.5, 2.4)	0.51	(0.28, 0.73)	0.98	-0.05	(-0.39, 0.28)	0.81
RobustMeanAbsoluteDeviation	3.4	(0.9, 12.3)	0.73	(0.53, 0.92)	0.048	0.44	(0.13, 0.68)	0.022
MeanAbsoluteDeviation	3.9	(1.0, 14.8)	0.76	(0.58, 0.95)	0.019	0.50	(0.18, 0.71)	0.008
TotalEnergy	1.1	(0.5, 2.3)	0.52	(0.29, 0.75)	0.91	-0.03	(-0.36, 0.30)	0.89
Maximum	2.0	(0.8, 5.0)	0.71	(0.50, 0.91)	0.068	0.34	(-0.02, 0.57)	0.088
RootMeanSquared	2.5	(1.0, 6.6)	0.74	(0.55, 0.94)	0.033	0.38	(0.03, 0.65)	0.053
90Percentile	2.8	(1.0, 7.6)	0.75	(0.55, 0.94)	0.029	0.41	(0.07, 0.67)	0.036
Minimum	0.4	(0.1, 1.4)	0.68	(0.46, 0.89)	0.13	-0.31	(-0.61, 0.05)	0.12
Entropy	3.0	(1.0, 8.7)	0.76	(0.57, 0.94)	0.022	0.47	(0.18, 0.68)	0.013
Range	2.3	(0.9, 6.0)	0.71	(0.51, 0.92)	0.061	0.36	(0.02, 0.60)	0.065
Variance	3.8	(1.2, 11.9)	0.80	(0.62, 0.97)	0.008	0.51	(0.23, 0.71)	0.007
10Percentile	1.7	(0.7, 4.0)	0.62	(0.40, 0.84)	0.30	0.20	(-0.18, 0.54)	0.31
Kurtosis	2.8	(0.7, 10.6)	0.66	(0.43, 0.90)	0.15	0.18	(-0.23, 0.53)	0.38
Mean	2.4	(1.0, 6.2)	0.74	(0.54, 0.93)	0.038	0.37	(-0.00, 0.66)	0.059
GLCM Features								
JointAverage	3.5	(1.2, 10.1)	0.78	(0.60, 0.96)	0.012	0.45	(0.12, 0.69)	0.017
SumAverage	3.5	(1.2, 10.1)	0.78	(0.60, 0.96)	0.012	0.45	(0.12, 0.69)	0.017
JointEntropy	2.6	(0.9, 7.0)	0.72	(0.52, 0.92)	0.054	0.39	(0.09, 0.61)	0.042
ClusterShade	0.2	(0.0, 0.8)	0.80	(0.62, 0.97)	0.008	-0.50	(-0.74, -0.15)	0.008
MaximumProbability	0.6	(0.2, 1.3)	0.67	(0.46, 0.88)	0.14	-0.34	(-0.61, 0.01)	0.086
Idmn	1.0	(0.5, 2.2)	0.52	(0.29, 0.75)	0.91	0.03	(-0.32, 0.37)	0.87
JointEnergy	0.5	(0.2, 1.1)	0.68	(0.47, 0.89)	0.12	-0.33	(-0.59, -0.01)	0.09
Contrast	3.9	(0.7, 21.4)	0.71	(0.51, 0.91)	0.068	0.40	(0.07, 0.64)	0.040
DifferenceEntropy	2.5	(0.9, 6.9)	0.69	(0.49, 0.90)	0.094	0.37	(0.03, 0.61)	0.061
InverseVariance	0.5	(0.2, 1.2)	0.68	(0.47, 0.88)	0.13	-0.35	(-0.59, -0.04)	0.071
DifferenceVariance	5.1	(0.8, 32.5)	0.75	(0.55, 0.94)	0.029	0.43	(0.11, 0.67)	0.025
Idn	1.2	(0.5, 2.5)	0.52	(0.29, 0.75)	0.87	0.08	(-0.28, 0.42)	0.70
Idm	0.5	(0.2, 1.2)	0.65	(0.44, 0.87)	0.19	-0.32	(-0.56, -0.01)	0.10
Correlation	3.1	(1.0, 9.6)	0.74	(0.54, 0.93)	0.038	0.36	(-0.00, 0.64)	0.068
Autocorrelation	3.6	(1.2, 10.4)	0.79	(0.61, 0.97)	0.011	0.46	(0.13, 0.69)	0.016
SumEntropy	2.8	(1.0, 8.0)	0.74	(0.55, 0.93)	0.033	0.44	(0.15, 0.64)	0.023
MCC	2.9	(1.0, 8.4)	0.70	(0.49, 0.91)	0.085	0.35	(-0.02, 0.67)	0.075
SumSquares	3.6	(1.2, 11.1)	0.79	(0.61, 0.96)	0.011	0.48	(0.20, 0.67)	0.012
ClusterProminence	4.9	(1.5, 16.5)	0.84	(0.69, 0.99)	0.002	0.51	(0.24, 0.68)	0.007
Imc2	2.7	(1.0, 7.6)	0.73	(0.53, 0.92)	0.048	0.34	(-0.01, 0.64)	0.083
Imc1	0.7	(0.3, 1.5)	0.58	(0.35, 0.80)	0.52	-0.11	(-0.48, 0.26)	0.60
DifferenceAverage	2.6	(0.8, 8.4)	0.69	(0.49, 0.90)	0.094	0.38	(0.06, 0.63)	0.049
Id	0.5	(0.2, 1.2)	0.66	(0.45, 0.88)	0.15	-0.34	(-0.59, -0.01)	0.085
ClusterTendency	3.7	(1.2, 11.4)	0.79	(0.61, 0.96)	0.011	0.48	(0.20, 0.67)	0.012

Abbreviations: AUC = area under the receiver operating characteristic curve; CI = confidence interval; GLCM = gray-level contrast matrix; OR = odds ratio; *Change per 1-SD increase in variable; †Spearman's rank correlation coefficient.

Supplementary Table 4. Univariable associations of radiomic texture features (GLDM, GLRLM, GLSZM, and NGTDM) with MVD, based on both categorical and continuous MVD assessments.

	High MVD vs. Low MVD					Correlation with Continuous MVD		
GLDM Features	OR*	(95% CI)	AUC	(95% CI)	P	Rho†	(95% CI)	P
GrayLevelVariance	3.8	(1.2, 11.9)	0.80	(0.62, 0.97)	0.008	0.51	(0.23, 0.71)	0.007
HighGrayLevelEmphasis	3.5	(1.2, 10.4)	0.79	(0.61, 0.97)	0.011	0.46	(0.13, 0.69)	0.016
DependenceEntropy	3.3	(1.1, 9.7)	0.74	(0.55, 0.93)	0.033	0.34	(0.00, 0.60)	0.086
DependenceNonUniformity	0.8	(0.4, 1.8)	0.54	(0.31, 0.78)	0.72	-0.16	(-0.48, 0.17)	0.41
GrayLevelNonUniformity	0.6	(0.2, 1.8)	0.63	(0.40, 0.85)	0.28	-0.28	(-0.60, 0.08)	0.15
SmallDependenceEmphasis	1.9	(0.8, 4.7)	0.66	(0.45, 0.88)	0.15	0.33	(-0.02, 0.60)	0.089
SmallDependenceHighGrayLevelEmphasis	3.1	(1.1, 8.3)	0.77	(0.59, 0.96)	0.014	0.45	(0.12, 0.67)	0.018
DependenceNonUniformityNormalized	1.6	(0.7, 3.7)	0.60	(0.37, 0.83)	0.40	0.23	(-0.12, 0.53)	0.24
LargeDependenceEmphasis	0.7	(0.3, 1.5)	0.61	(0.38, 0.84)	0.35	-0.25	(-0.54, 0.10)	0.21
LargeDependenceLowGrayLevelEmphasis	0.4	(0.1, 1.1)	0.74	(0.55, 0.94)	0.033	-0.38	(-0.65, -0.01)	0.049
DependenceVariance	0.8	(0.4, 1.7)	0.58	(0.34, 0.81)	0.52	-0.19	(-0.51, 0.15)	0.33
LargeDependenceHighGrayLevelEmphasis	5.0	(1.1, 23.6)	0.77	(0.57, 0.96)	0.017	0.43	(0.03, 0.69)	0.027
SmallDependenceLowGrayLevelEmphasis	0.7	(0.3, 1.6)	0.59	(0.37, 0.82)	0.43	-0.15	(-0.50, 0.23)	0.46
LowGrayLevelEmphasis	0.5	(0.2, 1.2)	0.71	(0.50, 0.92)	0.068	-0.33	(-0.65, 0.06)	0.089
GLRLM Features								
ShortRunLowGrayLevelEmphasis	0.5	(0.2, 1.2)	0.69	(0.48, 0.90)	0.094	-0.30	(-0.62, 0.10)	0.12
GrayLevelVariance	3.9	(1.2, 12.6)	0.80	(0.63, 0.98)	0.007	0.52	(0.23, 0.72)	0.005
LowGrayLevelRunEmphasis	0.5	(0.2, 1.2)	0.70	(0.50, 0.91)	0.076	-0.33	(-0.64, 0.07)	0.095
GrayLevelNonUniformityNormalized	0.4	(0.1, 1.0)	0.73	(0.53, 0.92)	0.048	-0.43	(-0.67, -0.12)	0.024
RunVariance	0.7	(0.3, 1.5)	0.59	(0.36, 0.82)	0.46	-0.22	(-0.52, 0.13)	0.27
GrayLevelNonUniformity	0.6	(0.2, 1.7)	0.63	(0.40, 0.85)	0.28	-0.29	(-0.61, 0.08)	0.14
LongRunEmphasis	0.6	(0.3, 1.4)	0.60	(0.38, 0.83)	0.38	-0.26	(-0.54, 0.10)	0.20
ShortRunHighGrayLevelEmphasis	3.5	(1.2, 10.0)	0.79	(0.61, 0.97)	0.011	0.46	(0.13, 0.69)	0.015
RunLengthNonUniformity	0.8	(0.4, 1.8)	0.55	(0.32, 0.78)	0.68	-0.17	(-0.48, 0.17)	0.41
ShortRunEmphasis	1.7	(0.7, 3.8)	0.63	(0.41, 0.85)	0.26	0.29	(-0.06, 0.56)	0.14
LongRunHighGrayLevelEmphasis	3.7	(1.2, 11.0)	0.83	(0.65, 1.00)	0.003	0.51	(0.16, 0.73)	0.007
RunPercentage	1.6	(0.7, 3.6)	0.63	(0.40, 0.85)	0.28	0.28	(-0.06, 0.56)	0.15
LongRunLowGrayLevelEmphasis	0.4	(0.2, 1.1)	0.72	(0.52, 0.92)	0.054	-0.35	(-0.65, 0.04)	0.075
RunEntropy	3.9	(1.2, 13.2)	0.80	(0.62, 0.97)	0.008	0.50	(0.22, 0.69)	0.008
HighGrayLevelRunEmphasis	3.5	(1.2, 10.2)	0.78	(0.60, 0.96)	0.012	0.46	(0.13, 0.69)	0.016
RunLengthNonUniformityNormalized	1.7	(0.7, 3.8)	0.63	(0.41, 0.85)	0.26	0.29	(-0.06, 0.56)	0.14
GLSZM Features								
GrayLevelVariance	5.0	(1.4, 18.3)	0.82	(0.66, 0.99)	0.003	0.50	(0.20, 0.69)	0.008
ZoneVariance	0.5	(0.1, 2.8)	0.63	(0.41, 0.85)	0.26	-0.31	(-0.63, 0.08)	0.12
GrayLevelNonUniformityNormalized	0.2	(0.1, 0.8)	0.79	(0.62, 0.97)	0.009	-0.48	(-0.67, -0.19)	0.011
SizeZoneNonUniformityNormalized	3.6	(1.2, 10.8)	0.78	(0.60, 0.96)	0.012	0.45	(0.12, 0.70)	0.019
SizeZoneNonUniformity	0.9	(0.4, 2.0)	0.50	(0.27, 0.73)	>0.99	-0.08	(-0.40, 0.24)	0.71
GrayLevelNonUniformity	0.8	(0.3, 1.7)	0.60	(0.37, 0.83)	0.40	-0.24	(-0.55, 0.10)	0.22
LargeAreaEmphasis	0.5	(0.1, 2.8)	0.63	(0.41, 0.85)	0.26	-0.31	(-0.63, 0.08)	0.12
SmallAreaHighGrayLevelEmphasis	3.6	(1.2, 10.6)	0.79	(0.61, 0.97)	0.011	0.45	(0.12, 0.68)	0.018
ZonePercentage	1.8	(0.8, 4.4)	0.64	(0.42, 0.87)	0.22	0.30	(-0.06, 0.59)	0.13
LargeAreaLowGrayLevelEmphasis	0.5	(0.2, 1.1)	0.68	(0.47, 0.88)	0.13	-0.39	(-0.69, 0.01)	0.046
LargeAreaHighGrayLevelEmphasis	1.1	(0.5, 2.3)	0.50	(0.27, 0.73)	>0.99	-0.07	(-0.42, 0.31)	0.72
HighGrayLevelZoneEmphasis	3.4	(1.2, 9.7)	0.79	(0.60, 0.97)	0.011	0.45	(0.11, 0.70)	0.017
SmallAreaEmphasis	3.5	(1.2, 10.5)	0.77	(0.59, 0.96)	0.014	0.46	(0.12, 0.71)	0.017
LowGrayLevelZoneEmphasis	0.5	(0.2, 1.2)	0.66	(0.45, 0.88)	0.15	-0.28	(-0.61, 0.09)	0.15
ZoneEntropy	2.1	(0.8, 5.4)	0.68	(0.47, 0.89)	0.12	0.23	(-0.09, 0.50)	0.24
SmallAreaLowGrayLevelEmphasis	0.5	(0.2, 1.3)	0.63	(0.41, 0.85)	0.28	-0.21	(-0.54, 0.19)	0.30
NGTDM Features								
Coarseness	1.1	(0.5, 2.3)	0.59	(0.37, 0.82)	0.43	0.24	(-0.11, 0.56)	0.24
Complexity	2.4	(0.9, 6.0)	0.71	(0.51, 0.92)	0.061	0.34	(0.01, 0.59)	0.078
Strength	2.9	(0.9, 9.5)	0.73	(0.53, 0.93)	0.048	0.39	(0.00, 0.69)	0.042
Contrast	3.1	(0.9, 10.9)	0.67	(0.45, 0.89)	0.14	0.37	(0.00, 0.65)	0.056
Busyness	0.2	(0.0, 3.2)	0.71	(0.51, 0.91)	0.068	-0.38	(-0.70, 0.01)	0.050

Abbreviations: AUC = area under the receiver operating characteristic curve; CI = confidence interval; GLDM = gray-level difference matrix; GLRLM = gray-level run length matrix; GLSZM = gray-level size zone matrix; NGTDM = neighborhood gray tone difference matrix; OR = odds ratio;

*Change per 1-SD increase in variable;

†Spearman's rank correlation coefficient.
