# Supplementary material for: Dynamic contrast-enhanced breast MRI features correlate with invasive breast cancer angiogenesis
Source: NPJ Breast Cancer. 2021 Apr 16;7:42. doi: 10.1038/s41523-021-00247-3 (PMC8052427; doi:10.1038/s41523-021-00247-3)
Supplement: Supplementary file 2 — Reporting Summary [file 41523_2021_247_MOESM2_ESM.pdf]

nature research

Corresponding author(s): Savannah Partridge
Last updated by author(s): Oct 3, 2020

Reporting Summary

Nature Research wishes to improve the reproducibility of the work that we publish. This form provides structure for consistency and transparency in reporting. For further information on Nature Research policies, see our Editorial Policies and the Editorial Policy Checklist.

Statistics

For all statistical analyses, confirm that the following items are present in the figure legend, table legend, main text, or Methods section.

n/a	Confirmed	
☐	☒	The exact sample size (*n*) for each experimental group/condition, given as a discrete number and unit of measurement
☐	☒	A statement on whether measurements were taken from distinct samples or whether the same sample was measured repeatedly
☐	☒	The statistical test(s) used AND whether they are one- or two-sided<br>*Only common tests should be described solely by name; describe more complex techniques in the Methods section.*
☐	☒	A description of all covariates tested
☐	☒	A description of any assumptions or corrections, such as tests of normality and adjustment for multiple comparisons
☐	☒	A full description of the statistical parameters including central tendency (e.g. means) or other basic estimates (e.g. regression coefficient) AND variation (e.g. standard deviation) or associated estimates of uncertainty (e.g. confidence intervals)
☐	☒	For null hypothesis testing, the test statistic (e.g. *F*, *t*, *r*) with confidence intervals, effect sizes, degrees of freedom and *P* value noted<br>*Give P values as exact values whenever suitable.*
☒	☐	For Bayesian analysis, information on the choice of priors and Markov chain Monte Carlo settings
☒	☐	For hierarchical and complex designs, identification of the appropriate level for tests and full reporting of outcomes
☐	☒	Estimates of effect sizes (e.g. Cohen's *d*, Pearson's *r*), indicating how they were calculated

Our web collection on statistics for biologists contains articles on many of the points above.

Software and code

Policy information about availability of computer code

Data collection	The open source software platform 3D Slicer (v4.11.0 with pyradiomics library, www.slicer.org) was utilized for radiomics analysis as referenced in the methods section. Software for lesion segmentation and kinetics analysis were developed in-house using MATLAB (MathWorks,Natick, MA) and the open source software platform ImageJ (NIH, public domain, Bethesda, MD).
Data analysis	All data analysis was performed in R version 4.0.0. The following packages were used: boot (version 1.3-25), pROC (version 1.16.2), and forestplot (version 1.10).

For manuscripts utilizing custom algorithms or software that are central to the research but not yet described in published literature, software must be made available to editors and reviewers. We strongly encourage code deposition in a community repository (e.g. GitHub). See the Nature Research guidelines for submitting code & software for further information.

Data

Policy information about availability of data

All manuscripts must include a data availability statement. This statement should provide the following information, where applicable:

- Accession codes, unique identifiers, or web links for publicly available datasets
- A list of figures that have associated raw data
- A description of any restrictions on data availability

The datasets generated and/or analyzed during the current study (shown in Figs 1-4) are available from the corresponding author on reasonable request.

Field-specific reporting

Please select the one below that is the best fit for your research. If you are not sure, read the appropriate sections before making your selection.

☒ Life sciences ☐ Behavioural & social sciences ☐ Ecological, evolutionary & environmental sciences

For a reference copy of the document with all sections, see nature.com/documents/nr-reporting-summary-flat.pdf

Life sciences study design

All studies must disclose on these points even when the disclosure is negative.

Sample size	No prior sample size calculations were performed. All available subjects who met the inclusion criteria were included.
Data exclusions	Patients were excluded if they underwent treatment with surgery or chemotherapy prior to the MRI, as this could affect both the MRI and pathology features measured. Of 33 enrolled patients, six were excluded from the study due to either obscuration of MRI findings by post-biopsy changes (n=4) or unavailable pathologic (n=2) specimens.
Replication	Two pathologists interpreted the MVD and resolved differences through consensus.
Randomization	There was no randomization to groups. Due to the sample size, multivariable analysis was not performed. We did examine differences in patient and tumor characteristics between groups and no large imbalances were found.
Blinding	Pathologists interpreted the MVD blinded to all imaging and clinical information. The DCE-MRI images were analyzed blinded to the pathologic and clinical information.

Reporting for specific materials, systems and methods

We require information from authors about some types of materials, experimental systems and methods used in many studies. Here, indicate whether each material, system or method listed is relevant to your study. If you are not sure if a list item applies to your research, read the appropriate section before selecting a response.

Materials & experimental systems

n/a	Involved in the study	
☒	☐	Antibodies
☒	☐	Eukaryotic cell lines
☒	☐	Palaeontology and archaeology
☒	☐	Animals and other organisms
☐	☒	Human research participants
☐	☒	Clinical data
☒	☐	Dual use research of concern

Methods

n/a	Involved in the study	
☒	☐	ChIP-seq
☒	☐	Flow cytometry
☒	☐	MRI-based neuroimaging

Human research participants

Policy information about studies involving human research participants

Population characteristics	Our participants consisted of 33 women with a diagnosis of stage I - III invasive breast cancer, with median age 53 years (range 30 to 88).
Recruitment	Patients over the age of 18 were prospectively recruited for the study if they had a new diagnosis of stage I - III invasive breast cancer with a minimum lesion size of 1.5 cm identified on core needle biopsy and underwent a clinical breast MRI at SCCA to evaluate the extent of disease.
Ethics oversight	This prospective study was approved by the Institutional Review Board of the Fred Hutchinson Cancer Research Center (IR#8148) and was compliant with the Health Insurance Portability and Accountability Act.

Note that full information on the approval of the study protocol must also be provided in the manuscript.

Clinical data

Policy information about clinical studies

All manuscripts should comply with the ICMJE guidelines for publication of clinical research and a completed CONSORT checklist must be included with all submissions.

Clinical trial registration	*Provide the trial registration number from ClinicalTrials.gov or an equivalent agency.*

Study protocol	FHCRC IRB #8148
Data collection	This was a single institution study at the Seattle Cancer Care Alliance with patients recruited from January 2016 to July 2017. Data collection was completed from January 2016 to August 2020
Outcomes	Our primary outcome measure was to evaluate the association between microvessel density and semi-quantitative kinetic parameters on dynamic contrast enhanced breast MRI. Our secondary outcome measure involved investigating the association of radiomics features and microvessel density.
